# Perceptions of Sexual Abuse in Sport: A Qualitative Study in the Portuguese Sports Community

**DOI:** 10.3389/fspor.2022.838480

**Published:** 2022-06-23

**Authors:** Joana Alexandre, Catarina Castro, Maria Gama, Patrícia Antunes

**Affiliations:** ^1^Department of Psychology, Center for Psychological Research and Social Intervention - CIS-IUL, Iscte - University Institute of Lisbon, Lisbon, Portugal; ^2^Amadora Inova, Amadora, Portugal

**Keywords:** sexual abuse in sport, risk factors, victims, perpetrators, social perceptions

## Abstract

Child sexual abuse is a complex issue that can take place in different contexts. Sports settings have specific features which pose increased risk for sexual abuse to occur. Recently, a country-specific roadmap for effective child safeguarding in sport was launched. Considering the need to achieve a comprehensive picture of violence against children in sports settings in Portugal, we analyzed the perceptions of the sports community in Portugal regarding child sexual abuse, its victims and perpetrators, and the specific risk factors in sports settings, as studies about this specific topic are scarce at the national level. A descriptive exploratory study was conducted using an online questionnaire with open-ended questions. Three hundred participants, i.e., sports managers, coaches, and athletes over 18 years of age (*M* = 33.13; *SD* = 13.062), of which 55.7% were female, answered. A thematic analysis of these data was conducted using NVivo software. Inter-rater agreement was strong for almost all variables. Results indicated that sexual abuse is perceived as being associated with physical and emotional abusive behaviors for which there is no consent from the victim, in a relationship that is guided by a relationship with power imbalances. Victims were mainly perceived as being female children, and perpetrators as adult males in a powerful position over the victim. As to possible signs of sexual abuse victimization, results showed that the participants identify behaviors, such as isolation, and physical evidence, such as marks and injuries. Risk factors specific to sports setting included the physical contact involved in many modalities, as well as the close and trustful relationship established between coach and athlete. Results are in line with previous studies showing that coaches, athletes, and sports managers share a common understanding of sexual abuse, although not always accurate. These results shed light on important practical and policy implications relevant to country-specific sport policies for effectively safeguarding children.

## Introduction

There is considerable evidence among children and adolescents, regarding the psychological and social health benefits of participation in sports (e.g., Eime et al., 2013; Australian Royal Commission into Institutional Responses to Child Sexual Abuse, 2017; Neto and Nery, 2018). However, sports settings also encompass specific risks (Hartill, 2009) but the protection of children in sport has come to light long after many other social concerns, such as disability or race equity (Darling et al., 2020).

In fact, sports' contexts do not always promote overall wellbeing. Amongst other issues, violence in sport must be addressed (Hartill, 2009). Violence and abuse are considered pervasive global issues, with important negative consequences for victims (e.g., on emotional wellbeing, mental health, and internalizing behaviors), their families (e.g., damaging relationships, particularly when the perpetrator is a family member), and society. Violence against children, in concrete, includes, for example, maltreatment (e.g., physical), bullying (e.g., Marracho et al., 2021), psychological violence, or sexual abuse.

Recent data from the project “Child Abuse in Sport: European Statistics (CASES),” involving six European countries (Austria, Belgium, Germany, Romania, Spain, and the United Kingdom) (Hartill et al., 2021) showed that the most common experience of interpersonal violence against children under 18 in sport was psychological violence (65%), followed by physical violence (44%), neglect (37%), and non-contact sexual violence (35%); only 20% of participants reported contact sexual violence, which is in line with previous findings. For example, a study on interpersonal violence against children in sport in the Netherlands and Belgium (Vertommen et al., 2016) indicated a higher rate of psychological violence (38% reported at least one experience of this type of interpersonal violence), followed by physical violence (11%) and sexual violence (14%). Gender differences were also found in a German study (Ohlert et al., 2017), with female athletes indicating higher prevalence of severe sexual violence, compared to male athletes. This is true also for non-heterosexual athletes, i.e., they reported more frequent experiences of severe sexual violence. Other findings also showed that 38% of athletes have experienced at least one sexual violence situation (ranging from milder aggressions such as sexual remarks or sexist jokes, to severe sexual abuse such as sex with penetration; Ohlert et al., 2017).

Although sexual abuse seems to be less prevalent, evidence shows that the real prevalence tends to be higher than the reported rate, due to aspects such as the sensitivity of the topic, the methodological procedures in data collection, and the complex nature of the disclosure process (Bjørnseth and Szabo, 2018).

Considering a children's rights approach, and the growing number of children and youth federated in sports, it has become imperative to address the issue of child protection in sports setting.

Recently, the Portuguese 2021–2024 National Strategy for the Rights of the Child stressed the need to design a National Plan for children and youth in sports, and to create child safeguarding officers (CSO). This strategy is described as an “integrated and comprehensive approach to children's rights, based on five main pillars: Promoting wellbeing and equal opportunities for all children and young people; Supporting families and parenting; Promoting access to information and participation for children and young people; Preventing and combating violence against children and young people; and Promoting the production of tools and scientific knowledge to enhance a global vision of children and young people's rights”^1^. The first 2 years (2021–2022) are being focused on the first four pillars, with the goal of strengthening prevention and intervention strategies regarding intrafamily violence, i.e., training of professionals), as well as preventing and tackling all other forms of violence against children and young people or perpetrated by them. The approval of the National Plan for the Protection of Children and Young People in Sports, the implementation of a national study on violence against children to be carried out with participation of children themselves, and the qualification of interventions in the field of juvenile justice are also expected goals. A country-specific roadmap for effective child safeguarding was recently launched (Council of Europe, 2022) and includes as expected outcomes “the development of competences and skills of those who will have a role to play in implementing the roadmaps and setting up CSO roles” (p. 5), as well as opportunities for peer-learning and capacity building. The roadmap was tailor-made, but it was built under the joint European project “Child Safeguarding in Sport” (CSiS) that involves, besides Portugal, Austria, Belgium, Croatia, and Israel.

Thus, the development of prevention strategies and capacity building implies a comprehensive picture of violence against children in sports settings in Portugal, in concrete. Bullying, specifically, has deserved some attention at the national level. Nery et al. (2019) recently analyzed the incidence and nature of bullying behaviors in male adolescent athletes from nine different sport modalities, and 97 sport clubs across Portugal, providing some insights about prevalence and impacts. The same authors also provided anti-bullying guidelines based on the results of that research (Nery et al., 2020). Regarding other forms of violence, such as sexual abuse, studies are scarce at a national level.

Child sexual abuse is defined by the World Health Organization (WHO) as the “involvement of a child in sexual activity that he or she does not fully comprehend, is unable to give informed consent to, or for which the child is not developmentally prepared and cannot give consent, or that violates the laws or social taboos of society” (1999, p. 15). The perpetrator can be “an adult or another child who, by his/her age or development, is in a relationship of responsibility, trust, or power toward the victim” (World Health Organization, 1999, p. 15). The sexual activity can imply contact or non-contact acts (Mathews and Collin-Vézina, 2019), and is intended to gratify or satisfy the needs of the perpetrator (World Health Organization, 1999; Brackenridge et al., 2008; see also Hartill, 2009).

Sexual abuse in sport is mainly perpetrated by men, and usually by coaches or doctors (Stirling and Kerr, 2009; United Nations Office on Drugs Crime, 2021), and/or other adults working in sports facilities (Darling et al., 2020), although recent results suggest that fellow athletes may also be perpetrators (Bjørnseth and Szabo, 2018; Hartill et al., 2021). Typically, the perpetrator has a good reputation and the trust of parents and young athletes. Perpetrators take advantage of a male-dominated, power-imbalanced context, as well as positive, close, or intense relationships, to exert their power and dominance (Darling et al., 2020). Other conditions, specific to this context, have also been identified as risk factors (Ecorys and Vertommen, 2019), such as changing rooms, traveling away to competitions/overnight stays, or need for carpooling (e.g., coaches' car).

Available data consistently show that most victims are young female athletes (Brackenridge, 1994; Darling et al., 2020), although a significant proportion of boys are also victimized (Parent, 2012). This gender discrepancy can be due to two aspects: (1) fewer studies including boys in their sample, and (2) being harder for male athletes to disclose abuse because boys have more difficulty reporting sexual abuse experiences, despite fear of stigma and punishment being common for both genders and considered obstacles to disclosure (Leahy et al., 2008; Adami et al., 2017).

Despite the lack of data on victims of sexual abuse in sport, particularly in the Portuguese context, there has been a growing number of reports of sexual abuse toward children and adolescents worldwide (from 1 million in 2010, to 17 million in 2019, and 21.7 million in 2020). In the Portuguese context, according to the Observatory of Sexual Crime from the Portuguese Judicial Police (OCS/PJ), inquiries of sexual crimes also increased by 25% between 2019 and 2020. From the analysis of inquiry cases leading to prosecution between 2019 and the first half of 2021, children between eight and 13 years old (83.9% in 2019, 80.5% in 2020, and 78.9% by the end of the first half of 2021) were identified as the main victims, and a predominance of female victims (84% in 2019, 82% in 2020 and 88% by the end of the first half of this year) was also verified. This is in line with what is reported in other countries^2^. This adds to the importance of understanding how the sport's community perceives child sexual abuse characteristics, and the risk factors increasing the occurrence of sexual abuse. Also, child sexual abuse myths seem to legitimize abusive behaviors, usually related with higher levels of victim blame, and lower levels of offender liability (Chim et al., 2020). It is, thus, important to capture these perceptions, to better support prevention and intervention actions.

According to Hartill et al. (2021), athletes at high competition levels (26%) report more frequent experiences of child sexual violence in sport. Similar results were also found by Bjørnseth and Szabo (2018) and Vertommen et al. (2016): for high performing/elite and sub-elite athletes the pursuit of excellence at any cost is a more pressing reality (Australian Royal Commission into Institutional Responses to Child Sexual Abuse, 2017), with coaches prioritizing performance over the athletes' wellbeing (Wilinsky and McCabe, 2020). Other characteristics in sport increase the risk of sexual abuse, such as the need for physical touch, and hierarchical structures in sport management (Gaedicke et al., 2021). All these factors are exacerbated in the sports elite context, where the coach's power is not only felt over athletes, but also over their parents (Wilinsky and McCabe, 2020). Findings also suggest that children from minority groups are at a greater risk (Vertommen et al., 2016; Bjørnseth and Szabo, 2018).

At a macro level, it is important to stress that sports have been considered as settings with an institutional culture of physical abuse and bullying, that normalizes violence, harassment, and sexualized behaviors (Australian Royal Commission into Institutional Responses to Child Sexual Abuse, 2017). This illustrates the debate between the need to “keep a distance” as a measure to prevent (sexual) abuse in sport, and the need for supportive, close, trust-based relationships (Gaedicke et al., 2021). According to Roberts et al. (2019), power imbalance and isolation, along with social norms of tolerance, are conditions for sexual abuse to occur. The blind trust on coaches is usually reinforced by parents when entrusting their children to them for extended periods of time (e.g., internships for some weeks) (Ecorys and Vertommen, 2019). There is also a pervasive culture of silence, as child sexual abuse can destroy the reputation of sport modalities and institutions (Sanderson and Weathers, 2019).

Grooming is a key component of sexual violence in sports (Australian Royal Commission into Institutional Responses to Child Sexual Abuse, 2017; Bjørnseth and Szabo, 2018), which is often difficult to identify. Grooming can start with the coach giving the athlete a ride, but perpetrators also use sports-related rewards, such as allowing the athlete to play. The process of grooming is considered as a “psychological game” that varies between pressuring the athlete, punishing them, and rewarding them with gratification (Bjørnseth and Szabo, 2018), with the aim to isolate them. All these characteristics of sexual abuse are in line with the model of Roberts et al. (2019), that identified power imbalances, winner-take-all rewards and isolation as structural factors related to non-accidental violence in sport.

Considering the specificities of sports contexts and the implied interplay between individual, familial, contextual, and cultural variables, child sexual abuse disclosures are not easy, but they are crucial for preventing further sexual abuse victimization and supporting victims. Common barriers to victims' disclosure in sports settings are the fear of not being believed, fear of consequences (e.g., not be allowed to play), feelings of shame and embarrassment, uncertainty about what is abusive behavior and what is right or wrong, fear of negative impacts on future success (e.g., effects on the sporting career; Australian Royal Commission into Institutional Responses to Child Sexual Abuse, 2017).

One way to address sexual abuse in sports settings is to focus precisely on disclosure. There is strong evidence that disclosures “are more likely to occur in a dialogical context—formal helping relationships, as well as other relationships, such as peers and trusted adults. Providing information and education on topics of sexuality in general, and sexual abuse specifically, can help children and youth to disclose. Raising awareness and prevention programs can promote disclosures of sexual violence committed against children and youth” (Alaggia et al., 2017, p. 276). According to Ungar et al.'s (2009) study with young participants, the optimal conditions for disclosure of abuse involve having (1) someone directly asking them about their experiences of abuse, (2) someone who listens and responds appropriately, (3) available definitions of abuse and language to describe their experiences, (4) a sense of control over the process of disclosure (anonymity, confidentiality), (5) knowledge of the resources to get help, and (6) effective responses from adults to keep youth safe. Hartill et al. (2021) study on the prevalence of child abuse in sport in European countries concluded that respondents rarely report the experience of IVAC (Interpersonal Violence Against Children) to someone in sport, which derives, according to the authors, from respondents not knowing where to report in the context of sport, nor feeling encouraged to do so (Hartill et al., 2021). The process of disclosure may assume many forms—it can be verbal or non-verbal-, which increases the need to empower society to recognize signals (e.g., using paintings or drawings to disclose, or behavioral cues, such as heightened anxiety; Australian Royal Commission into Institutional Responses to Child Sexual Abuse, 2017).

Some studies still point to a lack of resources and knowledge on how to prevent and act on sexual abuse in sports (e.g., Alaggia et al., 2017). However, improvements have been made in the last years, particularly in the European context as a whole, and at a country level in particular, focusing on different types of violence in sports settings. Policy guidelines, capacity building materials (e.g., educational toolkits), and awareness raising measures (e.g., campaigns) are some examples (Ecorys and Vertommen, 2019). The Council of Europe (2021) (CoE) launched in 2014–2015, the “Pro Safe Sport” (PSS), 2 years after the Pro Safe Sport + (PSS+) together with the EU, which was renamed in 2018 as “Start to Talk Initiative,” focused on prevention and protection measures to stop child sexual abuse.

Despite all efforts to spread awareness, and to put in practice concrete preventive measures, child sexual abuse—in sports and elsewhere—is still taboo. As with other social issues, communities collectively make sense of complex subjects (Moloney et al., 2014), such as sexual violence (Castiglione et al., 2012), sexual abuse of power (Singleton et al., 2019) or others (e.g., human rights, climate change), through common discourse and social interactions. Regarding child sexual abuse, there is still a lack of studies in Portugal describing how child sexual abuse in sports settings is perceived by the community. Despite shared perceptions (Muehlenhard and Kimes, 1999), more or less commonly accepted—by both the society and the existing academic literature—regarding child sexual abuse, myths persist, comprising inaccurate beliefs. According to Cromer and Goldsmith (2010), the accuracy of perceptions about child sexual abuse influences how people respond and behave toward perpetration, for example. Those authors also state that understanding these perceptions are “essential aspects in preventing future cycles of understanding and misunderstandings and in establishing appropriate strategies for prevention and support” (p. 638).

This study aimed to gather information on the perceptions of Portuguese athletes, coaches, and sports managers about child sexual abuse, its victims and perpetrators, and particular risk factors in sports settings, with the purpose of advancing knowledge in the Portuguese sport context. The main research questions were: how do athletes, coaches and sports managers appropriate the concept of child sexual abuse? What is their understanding about the characteristics of victims and perpetrators? What kind of risk factors do they identify in the sport context?

A qualitative study can be considered a first step to achieve initial insights into child sexual abuse, as it allows to understand more about how child sexual abuse is described. Also, and considering the scoping literature review recently conducted by Gaedicke et al. (2021), more studies focused on coaches' experiences and on both perspectives (athletes and coaches) are needed.

## Methods

### Participants

A total of 300 participants over the age of 18 years (*M* = 33.13; *SD* = 13.062) participated in the study, of which 105 were female athletes (31.5%), 33 male athletes (9.9%), 60 female coaches (18.0%), 91 male coaches (27.3%), 10 male sports managers (3.0%) and three female sports managers (0.9%). Of the total sample, 44.3% identified as male and 55.7% as female (Table 1).

**Table 1:** Sociodemographic characteristics of participants.

**Sociodemographic characteristics**	**Full sample**	
	***n***	**%**
**Gender**		
Female	168	55.7
Male	132	44.3
**Role in sports context and gender**		
Female athletes	105	31.5
Male athletes	33	9.9
Female coaches	60	18.0
Male coaches	91	27.3
Female sports managers	3	0.9
Male sports managers	10	3.0

*N = 300. Participants' average age is 33.13 years old (SD = 13.062; Min =18, Max =70)*.

### Instruments

A questionnaire was developed for the purpose of this study to allow for a qualitative analysis of a fairly unexplored topic, giving researchers space to comprehensively explore meanings (Albudaiwi, 2017) behind the participants' perceptions of different aspects of sexual abuse in sports. The questionnaire included questions regarding socio-demographic information (e.g., age, gender, role within a sport organization—e.g., being coach, sport manager, athlete), and five open-ended questions: “What do you understand by sexual abuse?”; “Who do you think the victims are?”; “What signs can the victims of sexual abuse show?”; “Who are the perpetrators and what are their characteristics?”; “In the Sports context, do you consider there are specific risk factors that increase the likelihood of sexual abuse situations to occur?.” The questionnaire allowed to minimize the effects of the researcher on participants' responses, considering the delicate nature of the subject (Vala and Castro, 2013), as well as allowing for more in-depth answers regarding perceptions of several aspects of sexual abuse.

### Design

A qualitative research framework was used, to allow for a deeper insight on perceptions of sexual abuse in sport. Considering its complexity and sensitivity, instead of a face-to-face interview approach, a questionnaire with open-ended questions was administered, thus encouraging participants to provide greater detail in their answers.

### Procedures

#### Data Collection

The questionnaire was uploaded to Qualtrics platform (Qualtrics, Provo, UT) for online data collection, and then shared on social media (namely Facebook) to specific contacts from sports units targeting athletes, coaches, and sports managers^3^, using the snowball sampling technique (Vinuto, 2014) to collect data. Thus, the link to access the questionnaire was shared via Facebook, sharing it to the public in general, and specifically inviting those affiliated to a sports organization to participate. The link was also sent to sports institutions, asking to disseminate it among its members (or those of other known sports entities). The questionnaire was available in Portuguese, and its completion took between 20 and 30 min on average. Rigorous attempts to recruit participants were considered (e.g., providing information about the purpose of the study, trying to contact different stakeholders to help reaching participants, and statements at the very beginning of the questionnaire about ethical safeguarding, providing information on a voluntary participation).

Saturation is usually applied to purposive samples (Hennink and Kaiser, 2019). In the current study, saturation was achieved after 3 months after keeping the questionnaire online. All participants gave their consent voluntarily, and confidentiality of their identities was always ensured, following regular ethical procedures (American Psychological Association, 2002), despite the use of Facebook as a primary dissemination channel. Participants accessed the questionnaire via a Qualtrics link, which has no connection to Facebook accounts, and guaranteed all anonymity in the data collection process.

#### Data Analysis

In order to identify patterns of meaning across the qualitative dataset, a thematic analysis was conducted (Braun and Clarke, 2012), with the following steps: (1) familiarization with data (float reading of answers to the open questions); (2) drawing out important concepts from the reading for each question; (3) generating themes, sub-themes, and codes (initial coding); the portions coded were designated as register units; register units were defined as any piece of information from the data—sentences, phrases or words, as long as its meaning could be attributed to a theme, sub-theme or code; (4) theme search: codes were classified and grouped into potential themes to capture and summarize the essential qualities of the participants' accounts; (5) theme review; (6) defining and naming themes; (7) organization by five dimensions, taking into account the open-ended questions in the questionnaire—the main dimensions were theory-driven, whereas themes and sub-themes were data-driven. Data was coded in NVivo 12 software (Nowell et al., 2017).

To ensure the validity and reliability of the analytic process, data coding was subjected to a process of inter-rater agreement. Inter-rater agreement and reliability are important tools to minimize biases in the categorization process (Landis and Koch, 1977; Fonseca et al., 2007; Kottner et al., 2011; McHugh, 2012). The kappa statistics (Cohen's kappa coefficient) is frequently used to test inter-rater agreement and reliability in nominal data (Fonseca et al., 2007; McHugh, 2012; Xie, 2013). It can be defined as the proportion of agreement between the raters after removing the proportion of agreement due by chance (Matos, 2014). For ordinal variables, weighted Cohen's kappa allows to calculate the degree of agreement and consistency between evaluators, and it is a preferable measure when the database is classified as ordered categories (Fonseca et al., 2007; Kottner et al., 2011).

After the initial categorization process by the first coder, 10% (*n* = 38)^4^ of the data were randomly selected to be categorized by two other independent coders on IBM SPSS Software. Each rater categorized participants' answers for the 10% dataset. The categorization was based on the same original themes, sub themes and codes defined by the first researcher.

Data analysis and result reporting followed the main recommendations addressed by Tong et al. (2007): considering its main goals, a qualitative approach, particularly a descriptive exploratory approach was used (see also Harding and Whitehead, 2013), resulting in a thematic analysis of the data.

## Findings

### Inter-rater Reliability Analysis

Inter-rater reliability analysis resulted in an average agreement of 81% (*k* = 0.81). According to McHugh (2012), this is considered a strong level of agreement between the two evaluators. The evaluators were two of the authors, with experience on inter-rater reliability analysis. For question 1, kappa ranged from 0.27 to 1.00^5^; for question 2 kappa ranged from 0.68 to 0.83 (*p* < 0.001 for all items); for question 3 kappa ranged from.60 to 1 (*p* < 0.001 for all items); for question 4 ranged from 0.77 to 1.00 (*p* < 0.001 for all items), and for question 5, inter-rater agreement was 0.99 (*p* < 0.001 for all items).

### Thematic Analysis

A total of 356 participants took part in the online questionnaire. However, 56 respondents did not fully fill in the main information requested, failing to indicate their role in the sports organization or their gender. This led to their exclusion, resulting in a final sample of 300 participants, from which the following results derive.

Thematic analysis resulted in five dimensions, 18 themes, and 59 sub-themes identified. Results are presented by each dimension: *Meanings of* s*exual abuse, Victims, Victimization signs, Perpetrators and their characteristics*, and *Risk factors in sports settings*. Results for each dimension's themes and sub-themes are described.

#### Meanings of Sexual Abuse

Six themes, and 11 sub-themes emerged within this dimension (Table 2).

**Table 2:** Sexual abuse themes and sub-themes.

**Themes**	**Sub-themes**	**Codes**	**RU**
Consent	No consent		213
	Inability to consent		15
Abusive behavior	Physical	Sexual actions	192
		Physical and intimate interaction	110
		Violence and attacks on physical integrity	59
	Emotional	Verbal	39
		Psychological	31
Power imbalance	Age	Child	14
		Adult	2
		Elderly	1
	Status and power		15
	Physical	Strength	2
	Gender	Female vs. Male	3
Perpetrator's motives	Sexual satisfaction		7
Other forms of violence	Harassment		11
	Rape		5
Legal frame			6

*RU, Register Units*.

When asked about what sexual abuse is, participants tended to describe it as an abusive conduct or abusive behavior, both physical and emotional, occurring in a relationship with power imbalances, and to which there is no consent from the victim, with the aim of obtaining sexual satisfaction for the perpetrator (“Someone using their greatest physical or social power to subdue another person for sexual purposes,” Male athlete #5; “The approximation, attempt, or the act itself, of any sexual behavior against someone else's will,” Female coach #1).

##### Abusive Behavior

Regarding physical abusive behavior, references were found on all participants and linked to three main aspects: sexual activities (“Penetration, oral sex, masturbation,” Female coach #56; “act of sexual nature,” Female athlete #63), intimate and physical interactions without mutual consent (“Every kind of non-consented intimate contact,” Female coach #22), and violence and threats on physical integrity (“Assault to the physical or psychological integrity,” Male coach #34).

For emotional abusive behavior, references were also found on all groups of participants, and were related to verbal and psychological abuse (“Physical or verbal actions,” Female athlete #25; “Disrespected by another person in terms of personal space, or psychologically,” Female athlete #71).

Overall, a pattern of meaning was found, in which sexual abuse was perceived as implying a lack of consent from the victim (“Every non-consented or unwanted sexual practice,” Female coach #47).

##### Power Imbalance

Participants highlighted the fact that sexual abuse tended to occur between (at least) two people with a power imbalance between them, with the perpetrator taking advantage of their role over the victim: “Any inappropriate contact or even some sort of comment between coach and athlete,” Male coach #74; “Any kind of non-consented sexual act, or which takes advantage of some influential position,” Male coach #75. Power imbalance as part of sexual abuse was particularly mentioned by male coaches.

Power imbalance due to age differences (i.e., typically the victim being a child and the aggressor an adult) was also highlighted (“... a situation where a child or adolescent's sexuality is invaded for the sexual satisfaction of an adult or someone older,” Female coach #35), as well due to higher status or position within the sports organization (“When someone in a position of power or authority takes advantage of someone's trust and respect to involve them in non-consented sexual activities,” Male coach #9).

##### Other Aspects of Sexual Abuse Conceptualization

There were also some remarks on sexual abuse being associated with other forms of violence, such as harassment and rape (“When someone harasses, disrespects, rapes, or acts on another person without their agreement,” Female athlete #46), as well as a perception of sexual gratification as a driving force for perpetrators (“Using his influence with the purpose of being sexually satisfied,” Male coach #81). Lastly, although to a lesser extent, the existence of a legal framework for sexual abuse was mentioned especially by coaches and sports managers (“In our juridic system, it constitutes a crime, foreseen and punished according to the penal code,” Female athlete #60).

#### Victims

Three themes and 10 sub-themes emerged within this dimension (Table 3).

**Table 3:** “Victims” Dimension's themes and sub-themes.

**Themes**	**Sub-themes**	**RU**
Age	Children	154
	Youth	49
	Adults	9
	Elderly	6
Gender	Female	212
	Male	18
Risk factor**s**	Vulnerability	21
	Economic, social or psychological	10
	Physical	8
	Professional hierarchy	5

*RU, Register Units*.

##### Victim's Age and Gender

All groups of participants shared a perception of the victim as being most often female and a child (“Women and children,” Female coach #4); and, to a lesser extent, a young person (“Underaged people,” Male coach #13).

##### Victim's Risk Factors

References to victims' characteristics were mainly related to vulnerability—either in an unspecified way (“Vulnerable adults,” Female coach #3; “More fragile and dependent people,” Female athlete #56), or linked to economic, social, and psychological aspects (“They present emotional, social and/or psychological instability,” Female athlete #85; “Institutionalized people, from an underprivileged social class,” Female athlete #14).

#### Victimization Signs

Six themes and 19 sub-themes emerged within this dimension (Table 4).

**Table 4:** “Victimization Signs” dimension's themes and sub-themes.

**Themes**	**Sub-themes**	**RU**
Negative emotions	Fear	62
	Low self-esteem	23
	Anger	18
	Embarrassment	17
	Sadness	15
	Apathy	12
	Unsafety	8
	Guilt	3
Internalizing behavior	Isolation	54
	Shyness	13
Physical indicators	Physical marks	122
	Repulse to physical contact	20
	Pregnancy and illness	2
Psychopathological indicators	Depression	49
	Unspecified psychological symptoms	49
	Anxiety	9
Externalizing behavior	Behavioral changes	16
	Aggressiveness	11
Sexual behavior	Repulsion to physical contact	20
	Constraints in intimacy	15

*RU, Register Units*.

##### Negative Emotions

The predominance of negative emotions emerged from the dataset: fear was the most reported emotional sign (“Traumas and fear,” Male athlete #3), followed by low self-esteem, and feelings of anger, embarrassment, or sadness (“Low self-esteem, sadness, embarrassment…,” Female athlete #2).

##### Internalizing Behavior

Participants considered isolation (“Distancing and isolation,” Male coach #31) and shyness also as signs of possible sexual abuse victimization. Isolation was mentioned by all groups of participants.

##### Physical Indicators

Several references were made to victims displaying physical indicators when sexually abused. Physical marks (“Bleeding from the anal or genital orifices,” Male athlete #27; “skin marks,” Male coach #78; “bruises,” Female athlete #49) emerged on all groups.

##### Psychopathological Indicators

Respondents identified several psychological symptoms as signs of sexual abuse. Depression was consensually mentioned as an indicator, followed by unspecified general psychological signs (“Psychological scars,” Female athlete #8), and anxiety symptoms.

##### Externalizing Behavior

Other signs of victimization were shared by all groups of participants include observable behaviors, such as an overall change in the victim's daily behavior (“Behavioral deviations,” Male coach #18), and, more particularly, increased aggressiveness (“Irritable, aggressive,” Male coach #63).

##### Sexual Behavior Avoidance

Although to a lesser extent, participants referred changes in sexual behavior as an indicator of sexual abuse victimization, particularly the increased difficulty in intimacy with others (“Awkwardness in more intimate situations (e.g., nudity in changing rooms),” Female athlete #86). A repulsion to physical contact by the victims also emerged from the dataset (“Also discomfort with someone else's touch,” Female athlete #86).

#### Perpetrators and Their Characteristics

Two themes, 12 sub-themes and 11 codes emerged within this dimension (Table 5).

**Table 5:** “Perpetrators and their characteristics” dimension's themes, sub-themes and codes.

**Themes**	**Themes**	**Codes**	**RU**
Perpetrator's	Pathologizing	Pedophilia	61
characteristi cs		Mental illness	7
	Position of power		59
	Age	Adult	44
		Youth	5
	Proximity to the victim		32
	Physical and psychological	Violence/ Aggressiveness	20
	characteristics	Strength	
		History of violent behavior	13
		Manipulation	13
		Need for sense of superiority	7
Perpetrator's	Gender	Male	87
identity		Female	11
	Family member		33
	Any person		28
	Sports coaches		16
	Friend		9

*RU, Register Units*.

##### Perpetrators' Characteristics

Typically, perpetrators were perceived as taking advantage from their powerful position (“People with power over the victim,” Male coach #19; “People with positions at work or in the society that are more favorable than the victim's,” Female athlete #4) by all groups of participants, except sports managers. Compared to all other groups of participants, female athletes and coaches tended to pathologize the perpetrator (“Pedophiles or people with mental issues,” Female athlete #42), and to report that perpetrators are usually adults and physically strong (“Usually men, stronger than the victim,” Male athlete #28). Aggressiveness and violence were also perceived as perpetrators' characteristics (“In most cases, they are men, with a tendency to be violent,” Male athlete #1; “aggressive characteristics,” Female athlete #33), being associated with previous experiences of violence, either as a victim or as a perpetrator (“People who also suffered from aggressions in their childhood, which leads them to later practice the same activities of sexual abuse,” Female athlete #78). Respondents also identified a pattern of manipulative behavior (“They have as characteristic a manipulative personality,” Male coach #73) and the need for dominance (“They have the need to feel superior to others,” Female coach #46).

##### Perpetrators' Identity

All groups of participants stated that offenders are male. Most often offenders were perceived as someone close to the victim—especially family members or someone with privileged access to the victim, namely coaches or friends (“Adults close to children and youth,” Female coach #10; “family members,” Male coach #27; “In a sports setting, I would say it could be the coaches,” Female athlete #69).

#### Risk Factors in Sports Settings

Seven themes emerged within this dimension (Table 6).

**Table 6:** “Risk factors in sports settings” dimension's themes.

**Themes**	**RU**
Physical contact	22
Relationship of proximity	17
Changing rooms	9
Status and power	8
Families' trust in professionals	5
Body exposure	4
Isolation during away periods	4

*RU, Register Units*.

Aside from sports managers, all other groups of participants identified specific aspects from sports settings which may confer additional risk to sexual abuse occurrences: physical contact (“Because there is more physical contact in sports, the risk increases,” Female athlete #11), and relationships of proximity (“The close relations between coaches and athletes,” Female athlete #10; “They [coach and athlete] spend a lot of time together and they [coaches] know them [athletes] well,” Female athlete #86). To a lesser extent, changing rooms were also perceived as locations of increased risk (“There may be [a risk] for children in changing rooms,” Female athlete #20). Imbalanced power dynamics between coaches or other staff members and athletes were also reported (“Sport is highly hierarchic, it's in its nature. Some people naturally hold a lot of power over others,” Male athlete #5).

## Discussion

The goal of the current study was to explore perceptions of sexual abuse in the sports community in the Portuguese context, considering the current Portuguese National Strategy for the Rights of the Child (2021–2024) and the recent launch of the country-specific roadmap for effective child safeguarding sport policies. Due to a lack of studies on child sexual abuse at the national level, particularly at the sports level, we developed a qualitative study that provided some insights about coaches', sports managers', and athletes' perceptions on child sexual abuse in this particular context.

An open-ended online study helped us to obtain data from a significant number of respondents, with different roles in sports settings, while still examining the phenomenon in great depth. Those aspects can be seen as strengths of the current study.

Considered a public health problem worldwide, due to its devasting consequences on children and adolescents (Homma et al., 2012; Castro et al., 2019), sexual abuse is still a taboo (Monteiro and Moleiro, 2021) in many communities. Several cases in sports settings have come to light in recent years, suggesting a greater incidence of abuse in sport than what is publicly known and acknowledged, which, in turn, highlighted the need to address this issue urgently (United Nations Office on Drugs Crime, 2021).

Regarding perceptions of sexual abuse in the sport community, the absence of consent was an important aspect reported by participants. As Gaedicke et al. (2021) pointed out, in sport, there is a certain construction of consent, derived from closeness and blurred boundaries, particularly between coaches and athletes, which enhances its complexity, and limits the ability of an athlete in a position of dependence to consent.

Physical or emotional abusive behaviors, as well as sexual activities, were also reported as shared cognitions about sexual abuse. Such results show a limited knowledge and a narrow perception of what sexual abuse really is, based more on common sense than in reality. Current definitions include both touching and non-touching behaviors, involving -or not- penetration, and can comprise behaviors such as fondling, exposure to adult sexuality (Darling et al., 2020), or online sexual abuse, which has significantly increased during the COVID-19 pandemic, according to the Internet Watch Foundation^6^.

Participants' overall perceptions also included other forms of violence within the sexual abuse act, such as rape or harassment (Darling et al., 2020). Participants' perceptions do not include, however, knowledge about the process of grooming. Usually, abuse is preceded by grooming (Brackenridge and Fasting, 2005). Grooming can be (1) physical, such as inappropriate touching that appears to be legitimated (e.g., helping an athlete during a gymnastics training taking advantage of physical touch); (2) psychological, in which a coach may, for instance, frequently advise an athlete and their parents that they need to spend more time with them in order to further improve (i.e., special individual training); or (3) it can happen in the social environment or in the community when, for example, a coach builds such a good reputation for competitive success that legitimizes their behavior, even if abusive. As Brackenridge and Fasting (2005) pointed out, “in sport, grooming is facilitated by the gradual building of the athlete's trust in their coach (or other authority figure) because the coach offers the opportunity of achieving tangible outcomes, such as winning competitions or representative honors and medals. The grooming process also involves intangible rewards such as feelings of being special, high self-esteem, confidence, superiority and security” (p. 35). Additionally, some studies show a different grooming process depending on the gender of the victim: kissing and declarations of love are more frequent with female athletes, while male athletes experience more aggressive grooming behaviors, such as being shown pornographic footage (Gaedicke et al., 2021).

Results also show that children are depicted as the main victims of sexual abuse situations where the perpetrator holds power over them, which is in line with results from a recent review (Gaedicke et al., 2021). It was also commonly mentioned the victim as being female (Hartill, 2009; Adami et al., 2017), and underprivileged. However, the evidence shows that individuals from disadvantaged backgrounds are not necessarily the main victims of child sexual abuse, which means that this social construction of victimology does not match the evidence that victims are also from privileged backgrounds. This result is particularly relevant for prevention initiatives aiming to address the barriers to disclosure and reporting by victims (Collin-Vézina et al., 2013).

Participants, particularly female athletes, and coaches, tended to pathologize the perpetrator. Pathologizing offenders is a common shared cognition in other studies, concerning not only sexual offenders, but also perpetrators of domestic violence (Conde and Machado, 2010). Pereira et al. (2019), for example, found similar perceptions in a study with legal professionals. Also, the idea that the offender has previous experiences of violence, either as a victim or as a perpetrator, puts more emphasis on internal aspects of offenders rather on contextual variables (the risk factors in sports) or on how grooming occurs.

Participants' perceptions seem overall aligned with the literature when identifying signs and consequences on victims (Alaggia, 2010; Adami et al., 2017; Alexandre and Agulhas, 2018; American Psychology Association, 2018). Regarding the perception of victims' possible signs of sexual abuse, data were also aligned with other studies showing that victims of sexual abuse are likely to display increased aggressiveness, irritability and change in behavior—such as a decrease in academic performance (Alexandre and Agulhas, 2018), although those signs are not only exclusive of victims of sexual abuse.

Participants also shared a perception of physical evidence of sexual abuse on victims. These are often unspecified, but include bruises, bleeding, and injuries in the genital area. According to Fazenda (2010), these are part of society's shared belief that sexual abuse always leaves a physical/visible mark on the victim.

Regarding participants' perceptions of risk factors in sports settings, all participants appart from managers share common ideas: aspects such as sports settings that justify a greater physical contact (e.g., during training or sports events), relationships of closeness and power imbalance between victims and offenders, and changing rooms, are the main concepts that emerged from the data, which is in line with previous findings (Adami et al., 2017; American Psychology Association, 2018). Isolation during practice or during internship periods away was mentioned as a risk factor as well. This aspect relates to characteristics perpetrators seek in victims: less supervision and more isolation (Marques et al., 2019). These results highlight what a recent UNODC report stresses: “the perpetration of abuse in sport exists because of silence, complacency and continued abuse of power and positions of trust, with perpetrators feeling that they can act with impunity” (United Nations Office on Drugs Crime, 2021, p. 202).

### Limitations, Implications for Practice and Future Research

Despite the potential practical contributions of this study, its limitations are mainly related to data collection tools and processes. Firstly, since this questionnaire was disseminated online (particularly through Facebook), there was less control over who the participants were, and if they indeed currently belong to a sports institution as athletes, coaches, managers, or other staff members, despite our efforts in the dissemination. Furthermore, the questionnaire was available only in Portuguese language, and no information on the participants' nationality or native tongue was collected, which poses the risk of misinterpretation of the questionnaire's content, while also limiting the possible pool of participants. Nevertheless, we did not receive any email from participants asking for clarifications.

Further limitations regard the range of participants' age, since respondents were required to be over the age of 18. This was to ensure legal autonomy in providing consent to the collection of personal information, prompted by the strategy used to recruit participants (i.e., via online public sharing of the questionnaire). This stands, however, as a limitation because it implies not considering the perceptions and experiences of children and underaged youth, who are, as previously pointed out, the main victims of sexual abuse in sports. Further research must consider young athletes' perspectives on the matter, in order to provide a deeper understanding of this phenomenon, as stressed in the National Plan for the Protection of Children and Young People in Sports, also described previously.

This study analyzed perceptions of sexual abuse and its specificities among sports managers, coaches, and athletes. Although the study was only limited to athletes over 18 years old, overall results reflect a collective meaning-making process about this topic, showing hegemonic views of sexual abuse, their victims, and offenders.

Regarding risk factors in sports, respondents, except sports managers, commonly mentioned power imbalance and physical contact as two of the main risk factors. At the same time, our findings highlight some misconceptions about what sexual abuse is, and about some of the characteristics of victims and offenders. Overall, those results highlight some of the main points that need to be considered for future actions, particularly when planning country-based campaigns and other prevention initiatives (e.g., training coaches on this subject).

According to Ecorys and Vertommen (2019), sexual abuse prevention in sports organizations implies providing training for the sports administrators to enhance their competence in preventing and managing sexual abuse cases. Our results have started to be used in training sessions with coaches (e.g., conducted in collaboration with the National Observatory for Violence Against Athletes, ObNVA)^7^, for example, where accuracy (e.g., myths) and its implications (e.g., believing in victims, helping victims, providing appropriate support to victims following disclosure, protecting athletes) are one of the first topics. The goal is also to fill the knowledge gaps that need to be addressed among professionals.

Preventive measures inside sport organizations are meant to act upon external and internal barriers, such as measures related to the hiring of coaches and recruitment of volunteers, as well as management rules for the misbehavior of organization staff members toward the young people involved. In line with this model, the International Safeguards for Children in Sport (Rhind et al., 2014) defines eight safeguarding measures: (1). Developing policy; (2). Procedures for responding to safeguarding concerns; (3). Advice and support; (4). Minimizing risks to children; (5). Guidelines for behavior; (6). Recruiting, training, and communicating; (7). Working with partners, and 8. Monitoring and evaluating. Also, the International Olympic Committee (2021)^8^ developed an online tool for safeguarding athletes from harassment and abuse, which aims to be a five-section course: (1). What is harassment and abuse; (2). Recognizing the signs; (3). Case study; (4). Understanding your role, and (5). What can organizations do?, which includes the guidance from a victim of abuse who also shares their testimony.

In Europe, some countries already implement guidelines for policies and practices that prevent sexual abuse in sport. For instance, since 1996, the Netherlands Olympic Committee and Dutch Sports Confederation (NOC^*^NSF) have been developing policies and practices to prevent sexual intimidation, and in 2012 developed the “Sexual Intimidation Policy” toolkit. The Netherlands also developed a political framework for safe and respectful sports settings, involving its Ministry of Health, Welfare and Sport. In the Czech Republic, there is a guide to prevent sexual harassment in sport, and in Ireland, there is a manual for ethical behavior and good practices for children in sport (Mergaert et al., 2016). Outside Europe, Australia has in place a National Strategy to Prevent and Respond to Child Sexual Abuse (2021–2030), that addresses the issue of children in sport (Commonwealth of Australia, Department of the Prime Minister and Cabinet, 2021), as well as the “Play by the Rules” project as an online tool to educate about discrimination, sexual harassment, and the protection of children in sport^9^.

Regarding the Portuguese context, as we described elsewhere, the “Child Safeguarding in Sport” (CSiS) project aims to guide and accompany some European countries—namely Portugal—in the development of policies and practices that ensure a safe, positive, and empowering sports setting for all children. With the following process underlying the project—Design of country-specific roadmaps; Extension, redesign and update of the Pro-safe sport, an online resource center; Training seminars; and Setting up the European network of Child Safeguarding Officers –, results from the current study can inform some of the steps of this project and contribute to a more comprehensive implementation of preventive measures for child abuse in sports settings, which considers the perceptions of different members of the sports community concerning this problematic.

Regarding prevention and research, it is necessary to maintain a vision of sport as a system, involving all elements of this community in the prevention and intervention process. In this line of research, it is important to give voice to coaches, athletes (younger and older), sports directors and sports organizations to identify motivators and incentives (Yore et al., 2018). Giving voice to athletes could be a way of empowering them and giving them responsibility to own their destinies (Brackenridge, 2008). Also, it is crucial to identify and share measures to assess sexual violence in sport-specific settings (Yore et al., 2018), and evaluate the efficacy of educational interventions and initiatives (Brackenridge, 2008). Research on the circumstances facilitating protective factors in sport is paramount (Yore et al., 2018), as well as “the relationship between sport prevention policies and perceived security in sport among athletes and parents, the degree of political will among sport administrators to implement prevention policies, the potential for prevention to impede or enhance sports coaching at elite levels,” as suggested by Brackenridge (2008, p. 8). The 2021 Olympics brought to the media and to public awareness the theme of mental health and sexual abuse in sports with the voice of athletes such as Simone Biles, which reinforced to the general public the need to protect, prevent, and intervene in sports settings.

Sports play a large positive role in local communities and in the wellbeing and health of many of its members worldwide, particularly children and youth. However, sports settings also convey characteristics that may facilitate the perpetration of sexual abuse. As such, it is of the utmost importance to better understand this phenomenon considering sports settings' specificities, capacitating organizations and individuals to effectively prevent abusive behavior and promote the best out of sports activities.

## Data Availability Statement

The raw data supporting the conclusions of this article will be made available by the authors, without undue reservation.

## Ethics Statement

Data was collected when the ethical commitee of ISCTE was created; nevertheless ethical procedures were guaranteed, following APA ethical guidelines and the ethical guidelines of the Portuguese Psychologists Association (OPP); Participants provided their written informed consent to participate in this study.

## Author Contributions

JA structured this article, wrote the Introduction and Discussion sections, and overall supervised the entirety of the process. CC was part of the interrater agreement, wrote the Results section, and reviewed this article overall. MG collected data, cleaned the datasets, and was responsible for data initial coding. PA contributed as part of the interrater agreement, final thoughts and discussion, and the overall structure of this article. All authors contributed to the article and approved the submitted version.

## Conflict of Interest

The authors declare that the research was conducted in the absence of any commercial or financial relationships that could be construed as a potential conflict of interest.

## Publisher's Note

All claims expressed in this article are solely those of the authors and do not necessarily represent those of their affiliated organizations, or those of the publisher, the editors and the reviewers. Any product that may be evaluated in this article, or claim that may be made by its manufacturer, is not guaranteed or endorsed by the publisher.

## Footnotes

^1^ https://www.cnpdpcj.gov.pt/documents/10182/43525/National±Strategy±2021-2024_Plan±2021-2022/f570a1bf-b03d-43b3-8c8a-dfd3fd586ba7

^2^ https://epthinktank.eu/2021/11/16/european-day-on-the-protection-of-children-against-sexual-exploitation-and-sexual-abuse/

^3^Participants in the sports environment were encouraged to respond: “We would like only people who are connected to this context to participate: athletes over the age of 18, coaches, doctors, physical therapists, or other professionals who work in this context.”

^4^61% coaches, 29% athletes, 3% doctors, 8% other functions in sport organization.

^5^For question 1, the value of p-value was.000 (*p* < .001), except for the item Physical Behavior (*p* =.001) and Physical Power (*p* =.009).

^6^ https://annualreport2020.iwf.org.uk/

^7^ https://www.ismai.pt/pt/investigacao/obnva

^8^ https://olympics.com/athlete365/courses/safeguarding/

^9^ www.playbytherules.net.au
